# Predicting the Water Rebound Effect in China under the Shared Socioeconomic Pathways

**DOI:** 10.3390/ijerph18031326

**Published:** 2021-02-01

**Authors:** Aijun Guo, Rong Zhang, Xiaoyu Song, Fanglei Zhong, Daiwei Jiang, Yuan Song

**Affiliations:** 1 School of Economics, Lanzhou University, Lanzhou 730000, China; guoaj@lzu.edu.cn (A.G.); zhangrong19@lzu.edu.cn (R.Z.); jiangdw18@lzu.edu.cn (D.J.); 2 Institute of County Economic Development, Lanzhou University, Lanzhou 730000, China; 3 Institute of Rural Revitalization Strategy, Lanzhou University, Lanzhou 730000, China; 4 Northwest Institute of Eco-Environment and Resources, Chinese Academy of Sciences, Lanzhou 730000, China; 5 Gansu Meteorological Information and Technical Equipment Support Center, Lanzhou 730020, China; 13993123009@139.com

**Keywords:** water use efficiency, total water use, shared socioeconomic pathways, rebound effect

## Abstract

The rebound effect exists widely in the fields of energy, irrigation, and other resource utilizations. Previous studies have predicted the evolution of different resource utilizations under the shared socioeconomic pathways (SSPs), but it is still unclear whether total water use has a rebound effect. This study uses the SSPs as the basic prediction framework and evaluates the water resources and economic status of the provinces in China using the hydro-economic (HE) classification method. Then, combined with the SSPs scenario setting parameters, the conditional convergence model and the method recommended by the Food and Agriculture Organization of the United Nations (FAO) are used to simulate the changes in water use efficiency of the different provinces in China under different scenarios. Based on the future GDP forecast data of China’s provinces, combined with the forecast of water use efficiency changes, the total water use changes in China’s 31 provinces under different pathways from 2016 to 2030 are calculated. Among them, the future GDP data is predicted based on the Cobb–Douglas production function and SSPs scenario settings. Using a comprehensive evaluation of the evolution of the efficiency and the total amount, this study reveals whether there is a rebound effect. The results showed that with the continuous growth in the water use efficiency, the total water use had a “U” type trend, which indicated that there was a rebound effect in the total water use of China under the different SSPs. Based on this information, this study proposes some suggestions for irrigation water-saving technologies and policies.

## 1. Introduction

The “rebound effect” is a concept used in energy research that can help us more clearly quantify the impact of water productivity on water use [1]. The rebound effect was first proposed by Jevons [2]. He found that more efficient steam engines not only reduced coal consumption, but also led to a drop in coal prices, which ultimately increased the demand for coal (“Jevons’ Paradox”). The positive impact of energy efficiency on energy conservation is being questioned in the economic world [3,4]. Moreover, research in the field of water resources has found that the agricultural water supply also has a rebound effect [5,6,7,8]. With improvements in local small-scale efficiencies, this often leads to an expansion of the resource utilization scale, which leads to an increase in resource consumption at a larger scale. In more precise terms, the ultimate result of the practice of reducing the resource consumption per unit output and then reducing the total resource utilization through technological progress is often very complicated [9].

China has been short of water resources for a long time. The per capita water resources amount is 2200 m^3^, which is only one-fourth of the world average [10]. Moreover, the spatial and temporal distribution of water resources is uneven, and the supply and demand are not balanced among the regions. Hence, the sustainable utilization of water resources is a very serious issue. In 2012, the Chinese government issued opinions on the implementation of the strictest water resource management system, which establishes three red bottom lines: water resource development and utilization control, water use efficiency control, and water function area limitations for pollution absorption. Two of the red bottom lines are that the total water use of China will be controlled within 7000 × 10^8^ m^3^ by 2030, and the water use efficiency will reach or approach the world advanced level. In recent years, highly efficient irrigation and industrial water recycling technologies have led to a decline in water use intensity in most areas of China. The growth of water use in China is likely to continue to slow down, but uncertainties and potential water shortage problems still exist [11]. China’s land agency is rapidly transitioning to large-scale agriculture through the farmland transfer system released in 2014 [12]. In addition, there will be the adoption of water-conservation irrigation planning to cover 75% of the irrigated area by 2030 [11]. This may cause farmers to switch to water-intensive crops or expand their irrigated area, thereby offsetting the water savings due to future improvements in irrigation efficiency [13,14]. Except for Xinjiang, China’s arid and semiarid regions have adopted high industrial water recycling, which limits the potential for further water conservation. Moreover, the westward development of the industrial sector has also exacerbated water shortages in these regions. In addition, China’s urbanization is evolving at an unprecedented speed. Not only is the economy growing, but household wealth is also increasing [15]. The increase in per-capita income and the widespread supply of tap water will stimulate an increase in domestic water use [16,17]. Therefore, it is worth asking: can China achieve the most stringent water resources management objectives by 2030? Will there be a rebound effect in total water use after technological progress has led to improved water use efficiency? To answer these questions, it is necessary to predict the future water use efficiency and the total water use in China.

In the future socioeconomic scenario simulation field, the intergovernmental panel on climate change (IPCC) has proposed a framework for the prediction of shared socioeconomic pathways (SSPs). The five typical pathways are SSP1 (sustainability), SSP2 (middle of the road), SSP3 (fragmentation), SSP4 (inequality), and SSP5 (conventional development) [18,19,20]. It quantitatively describes the five typical developmental pathways of the social economy in the future, and it distinguishes the different emission concentrations caused by the different developmental pathways. In addition, it distinguishes the different climate change responses and adaptabilities formed by the different developmental pathways [21]. The prediction of social and economic scenarios under the different pathways is beneficial for the combination of social and economic development and the natural factor model. It can provide scientific support for the customization and implementation of a sustainable development strategy and provide a unified and comparable framework for many forecasting studies.

Therefore, this study utilizes the SSPs as the basic prediction framework. There have been many studies on SSPs in the field of resource and environment prediction [22]. Most of these studies are from the perspective of the impact and the risk of climate change, the impact of mitigation measures and other scenario applications, the coupling of different types of models, such as the integrated assessment model (IAM) [23], the hydrologic model [24], and the global climate model (GCM) (including the general circulation model) [25] to explore the problems of land use and water shortage on a global or national scale. Chinese scholars are primarily involved in the assessment of climate change impacts, such as water shortages [26,27] and drought disasters [28,29], and the estimation of basic elements of the SSPs such as population [30,31], GDP [32], urbanization [33], and land use [34,35]. At the global and regional scales, SSPs have been applied in the field of water resource predictions, which is an important scenario framework for water resource utilization. It provides a unified framework for water use prediction and can be directly linked with climate models [36,37,38]. Based on the original SSPs framework, the Water Futures and Solutions (WFAS) proposed the hydro-economic (HE) classification method. This method can be combined with the scenario settings of the SSPs to group regions according to their different economic and water resources conditions [36]. The extended SSPs-HE framework has obvious advantages and can more accurately set the optimal efficiency target value, convergence time, convergence speed, and other parameters for different regions. It can also predict the changes in water resource utilization by simulating the curves of changes in water resource utilization in the different regions under various scenarios. Previous studies have focused too much on small-scale water-saving effects and engineering fields, but the extended SSPs-HE framework combines a broad view of an entire hydrological basin with integrated water resources management, and evaluates the different social and economic pathways from the perspective of water use efficiency [21,36,39].

Therefore, this study uses the SSPs-HE framework to group the hydrological fields and economic management capabilities of 31 provinces in China to discuss how the water use efficiency of the different groups of provinces will develop under the five typical developmental pathways. Different from previous studies, this study simulates the dynamic evolution of China’s water use efficiency indicators under the theory of technological change, cites the conditional convergence model, and combines with the dynamic evolution of China’s economic development indicators that the team has. The purpose of this research is to obtain the dynamic evolution of water use and analyze in depth whether China’s total water use will have a rebound effect under the five typical developmental pathways.

The water use efficiency indicators of the Chinese government are primarily the water consumption per RMB 10,000 industrial added value and the effective utilization coefficient of irrigation water. However, due to the different characteristics of the different regions in China, there are great differences in the climate and precipitation and irrigation conditions [40,41]. These two indicators, especially the effective utilization coefficient of irrigation water, may not be applicable to all regions. For example, due to abundant rainfall in southern China, the irrigation system facilities are not more developed than that in the northern arid areas, but water use efficiency and output are not necessarily lower. From the perspective of operability and comparability, this study used the water use efficiency calculation index recommended by the Food and Agriculture Organization of the United Nations (FAO) [42]. The water use efficiency accounting index in this method assesses the impact of economic growth on the use of water resources, and can be closely integrated with the SSPs-HE framework. It involves all three sectors of agriculture, industry, and service, covering nearly all the water sectors in China.

Therefore, the overall principle of the simulation is to use the SSPs as the basis of the prediction framework, to classify the water resources and economic background of China’s provinces using the HE classification method, set parameters according to the regional characteristics and SSPs scenarios, and use the conditional convergence model and FAO’s method to simulate the changes in the water use efficiency of the different scenarios in the different provinces. Based on the future GDP forecast data of China’s provinces, combined with the forecast of water use efficiency changes, the total water use changes in China’s 31 provinces under different pathways from 2016 to 2030 are calculated. Among them, the future GDP data is predicted based on the Cobb–Douglas production function and the SSPs scenario settings. By comprehensively evaluating the evolution of water use efficiency and total water use, this study discusses whether China’s future water resource utilization will achieve the most stringent water resource management objectives and whether water resource consumption will have a rebound effect under the different socioeconomic developmental pathways.

## 2. Materials and Methods

**Figure 1:**
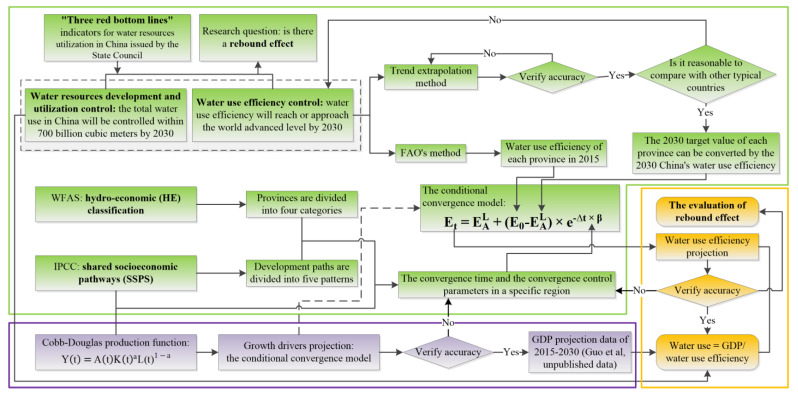
Flow chart of water use efficiency forecast and water use forecast for each province under the shared socioeconomic pathways (SSPs).

The overall research flow of this study is shown in Figure 1, which can be divided into three key parts. The first part is to simulate the evolution of water use efficiency in the future. The main core is the conditional convergence model, which utilizes water use efficiency from 2000 to 2017 as the historical basic data, and the data are from FAO [43]. Then, the trend extrapolation method was used to obtain China’s target water use efficiency in 2030. This was verified using other developed countries’ future water use efficiency evolution to ensure that the future target value of efficiency is reasonable and reliable [42]. For the problem of setting parameters for the different scenarios and different regional characteristics of future developmental pathways, this study used the SSPs scenario and the HE classification method to set the optimal efficiency targets and convergence times of the different provinces. The second portion regards the forecast of the total amount of water used in the future. This was primarily based on the forecast data of the future GDP that the team had, and it was calculated by dividing it with the future changes in water use efficiency in the region. The third portion of this study explored whether water use has a rebound effect by using a comprehensive assessment of the evolution of water use efficiency and the total water use. Among them, the future GDP data was predicted based on the Cobb–Douglas production function and the SSPs scenario settings. Each step of the simulation prediction method involved in this study was verified for accuracy.

### 2.1. The Calculation Method for the Water Use Efficiency in China’s Provinces during the Historical Period

The calculation of the water use efficiency in China’s provinces adopted the FAO’s method. This indicator is calculated as the sum of the three sectors of agriculture, industry, and service, and weighted according to the proportion of water used by each sector in total water use. It represents the value added per water used, expressed in USD/m^3^ of a certain economic sector [42], and the specific formula is as follows:

(1) $${\mathrm{WUE}\;=\;A}_{\mathrm{we}}{\;\times \;P}_{A}{\;+\;I}_{\mathrm{we}}{\;\times \;P}_{I}{\;+\;S}_{\mathrm{we}}{\;\times \;P}_{S},$$

where:

- WUE = water use efficiency (USD/m^3^);
- $A_{\mathrm{we}}$ = irrigated agriculture water use efficiency (USD/m^3^);
- $I_{\mathrm{we}}$ = industrial water use efficiency (USD/m^3^);
- $S_{\mathrm{we}}$ = services water use efficiency (USD/m^3^);
- $P_{A}$ = proportion of water used by the agricultural sector over the total use;
- $P_{I}$ = proportion of water used by the industrial sector over the total use;
- $P_{S}$ = proportion of water used by the service sector over the total use.

For the calculation of the agricultural water use efficiency, it was necessary to consider the ratio between the rainfed and irrigated yields in the area and deduct the output value of the rainfed agriculture. The specific calculation formula is as follows:

(2) $$A_{\mathrm{we}}\;=\;\frac{{\mathrm{GVA}}_{a}{\;\times \;(1\;-\;C}_{r})}{V_{a}},$$

where

- ${\mathrm{GVA}}_{a}$ = gross value added by agriculture (USD);
- $C_{r}$ = proportion of agricultural GVA produced by rainfed agriculture (%);
- $V_{a}$ = volume of water used by the agricultural sector (m^3^).

The proportion of agricultural GVA produced by rainfed agriculture was calculated according to the following formula in which the ratio between the rainfed and irrigated yields was calculated according to the United Nations recommended number 0.650 [42].

(3) $$C_{r}=\frac{1}{1+\frac{A_{i}}{\left(1-A_{i}\right)\times 0.650}},$$

where

- $A_{i}$ = proportion of irrigated land on the total cropland, in decimals.

The water use efficiency of industries and services was calculated as follows:

(4) $$I_{\mathrm{we}}\;=\;\frac{{\mathrm{GVA}}_{i}}{V_{i}},$$

(5) $$S_{\mathrm{we}}\;=\;\frac{{\mathrm{GVA}}_{s}}{V_{s}},$$

where

- ${\mathrm{GVA}}_{i}$ = gross value added by industrial (USD);
- $V_{i}$ = volume of water used by industrial (m^3^);
- ${\mathrm{GVA}}_{s}$ = gross value added by services (USD);
- $V_{s}$ = volume of water used by the service sector (m^3^).

### 2.2. Estimation and Verification Method of China’s Water Use Efficiency Target Value in 2030

In the future planning of water use efficiency goals, China has not set specific values, but mentioned that water use efficiency needs to reach or approach the world’s advanced level in 2030. This article used the water use efficiency index data of China and other countries from 2000 to 2017 [43], and the trend extrapolation method was used to obtain the water use efficiency level in 2030. This is a new indicator, with no pre-existing experience or data, so the primary interpretation rationale should be a comparison with the economic growth of the country. In addition, the indicator should, as a minimum, follow the same trend as the economic growth to be acceptable [42]. For verification and comparison, this study utilized the same method to estimate the water use efficiency of typical developed countries and developing countries in the world in 2030. Two typical countries were selected in this study, United States and Mozambique, to verify the reliability of the trend extrapolation.

### 2.3. The Conditional Convergence Model Method for Simulating the Evolution of Water Use Efficiency

Water use efficiency is in fact a manifestation of technological progress in the utilization efficiency of natural resources that has a direct impact on the total scale of natural resource utilization, which in turn can directly affect the sustainable development of regional social economies. The theory of technological change usually distinguishes two types of technological change: technological catch-up and technological diffusion. That is, the transmission of technology in advanced regions and the catch-up of technology in backward regions. At a certain stage of development, technological change will gradually converge to the optimal level of efficiency, this is a common opinion. Additionally, technologically backward regions improved faster than technologically advanced regions, and the reason is the distribution of the conditional convergence model. The conditional convergence model here is an exponential model, reflecting long-term changes in the technical efficiency of economies with similar structural characteristics [44,45,46,47].

The formula for predicting water use efficiency of the conditional convergence is as follows [48,49,50]:

(6) $$E_{t}{\;=\;E}_{A}^{L}{\;+\;(E}_{0}\;{-\;E}_{A}^{L}{)\;\times \;e}^{-\Delta t\;\times \;\beta },$$

where

- $E_{t}$ = water use efficiency in convergence time t (years);
- $E_{A}^{L}$ = water use efficiency for medium- to long-term (2030) targets;
- $E_{0}$ = initial (2015) water use efficiency in a region;
- Δt = time to convergence;
- β = the convergence control parameters in a specific region.

### 2.4. The SSPs-HE Framework Parameter Setting Method that Combines Regional Characteristics and Different Pathway Scenarios

The original SSPs framework described five typical global situations with different socioeconomic conditions, excluding water use scenarios [18,19,20]. Later studies added water use scenarios, especially the studies of Hanasaki and Wada [24,36]. Based on the existing studies, appropriate corresponding water use scenarios were formulated. Table 1 summarizes the key details of the SSPs framework for each water use scenario.

**Figure 2:**
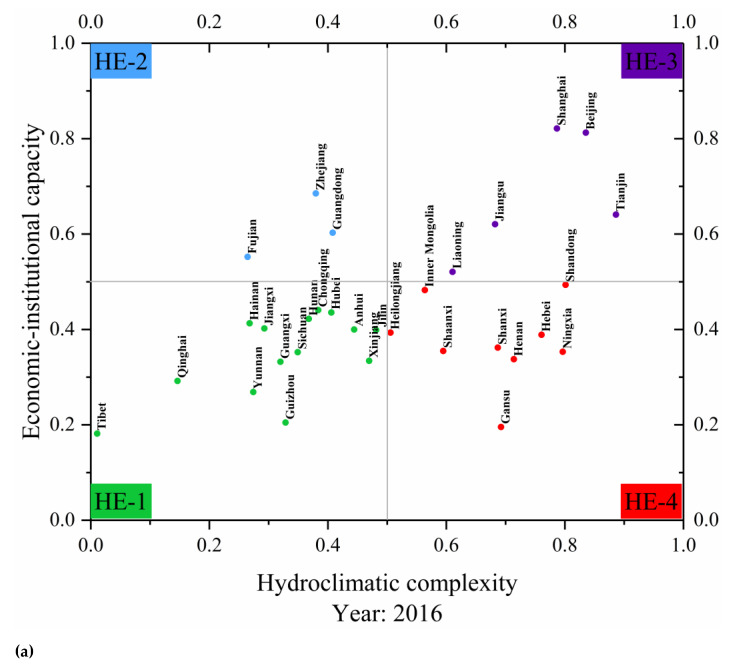

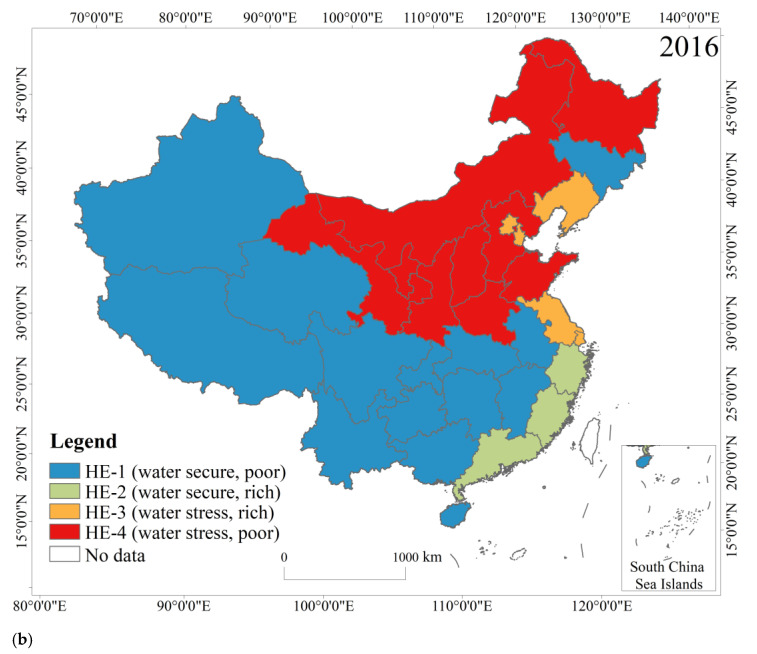
(**a**) Hydro-economic (HE) classification quadrants of the 31 Chinese provinces for 2016. (**b**) National distribution of HE classification for 31 Chinese provinces for 2016 [21].

The Water Futures and Solutions (WFAS) scenario proposed the HE classification method, this method can be combined with the scenario settings of the SSPs to group regions according to their different economic and water resources conditions. The HE classification method uses the calculation results of the research team in this study [21]. According to the HE classification method of the WFAS, the provincial administrative regions of China were classified (excluding the Hong Kong special administrative region, the Macao special administrative region, and the Taiwan region) (Figure 2). Among them, the economic–institutional coping capacity of the Y dimension was measured using the personal disposable income of each province in 2016 published by the National Bureau of Statistics, and the X dimension, which represents the hydroclimatic complexity, was measured using a comprehensive index converted from three indicators according to weights. The three indicators were the total water resources per capita, the ratio of annual total water withdrawal to the total water resources of each province, and the proportion of external water resources (from outside the regional boundaries) of the total water resources of each province [21].

According to the classification results of the HE, the following five socioeconomic development pathways were used to set different convergence targets, convergence speeds, and other parameters for provinces in the different quadrants of the HE.

### 2.5. Total Water Use Accounting

The calculation method of the total water use is the future GDP forecast data of the team divided by the future water use efficiency data of the region. For specific forecast methods, please refer to the literature [51].

(7) $$W_{\mathrm{ti}}=\;\frac{Y_{\mathrm{ti}}}{{\mathrm{WUE}}_{\mathrm{ti}}},$$

where

- $W_{\mathrm{ti}}$ = water use in year t of region i;
- $Y_{\mathrm{ti}}$ = GDP in year t of region i, (GDP data comes from the research team [51]);
- ${\mathrm{WUE}}_{\mathrm{ti}}$ = water use efficiency in year t of region i.

### 2.6. Data Sources

The historical water use efficiency accounting data of each province in China came from the National Bureau of Statistics [52]. The data of water use efficiency indicators for each country from 2000 to 2017 were obtained from FAO [43]. The GDP data of each country came from the World Bank [53], and China’s 2015–2030 GDP forecast data were obtained from this research team [51].

## 3. Simulation and Results Analysis

### 3.1. Forecast of China’s Water Use Efficiency Target Value in 2030

**Figure 3:**
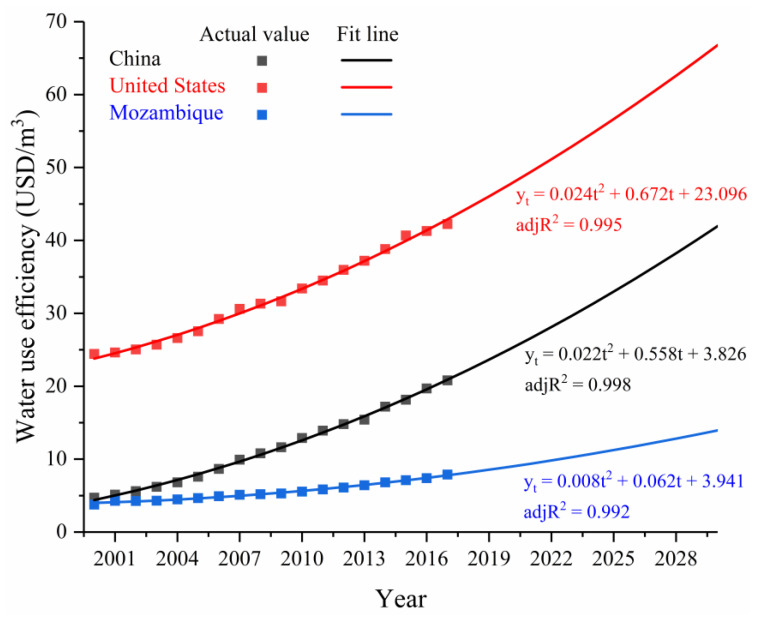
Actual value and fit line of the water use efficiency in various countries.

When the forecast object presents a certain upward or downward trend according to time changes, there is no obvious seasonal fluctuation, there is no jumping change in the development process of things, and a suitable function curve can be found to reflect this trend. This can be used as the trend extrapolation method for prediction. It can be seen from Figure 3 that the scatter plot of China’s water use efficiency indicators from 2000 to 2017 meets the conditions of trend extrapolation. After various function curves (e.g., exponential curve model, logarithmic curve model, polynomial curve model) fitting comparisons, the quadratic curve model was chosen, and the result was the best.

As shown in Figure 3, the model for China’s water use efficiency is “$y_{t}=0.022t^{2}+0.558t+3.826$”, where y_t_ is the water use efficiency index, and t is the time series (that is, the year 2000 is set to 1, the year 2001 is set to 2, and so on). In the model, Adjusted R^2^ (adj R^2^) = 0.998, F = 3520.6 > F_0.05_ (2,15), then the equation passes the significance test, and the fitting effect is very reliable.

**Figure 4:**
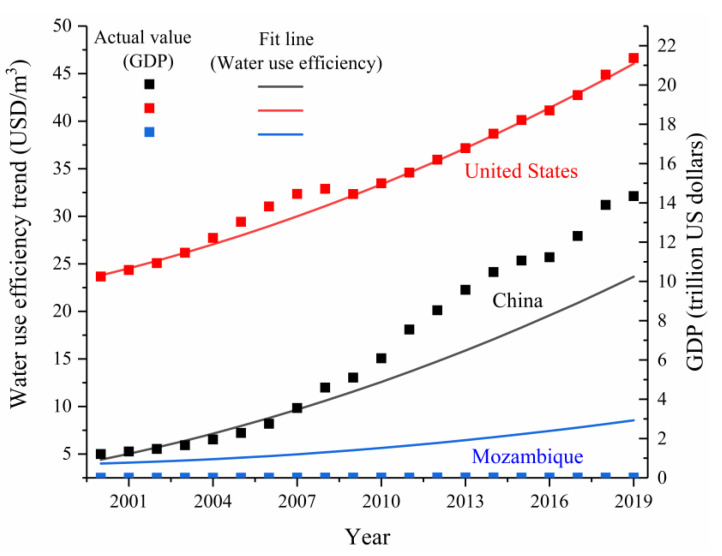
Fit line of the water use efficiency and the actual value of each country’s GDP.

The results showed that by 2030, China’s estimated water use efficiency level will be 42 USD/m^3^. In addition, it can be seen from Figure 4 that from 2000 to 2019, the growth trend of each country’s water use efficiency fitting line is consistent with the growth trend of the actual value trend line of GDP. Since this indicator should at least follow the same trend as economic growth before being accepted [42], it proves that the estimated model is more reliable. Additionally, the result of model fitting is very good, adjR^2^ values nearly all reached above 0.9 (Figure 3), and the F test values of U.S. and Mozambique water use efficiency estimation models were 1542.2 and 1006.1, respectively, so the equations passed the significance test. This proves that using the trend extrapolation method to estimate China’s water use efficiency level in 2030 is acceptable.

Although there was a certain gap between China’s water use efficiency of 42 USD/m^3^ and some developed countries’ water use efficiency levels in 2030, due to the actual national conditions of China’s water-saving irrigation and the gap compared to the water-saving technologies of developed countries in the world [41], the results were repeatedly compared. Therefore, it is believed that the result is reasonable and reliable. This study used this target efficiency as a benchmark and further converted it into the 2030 water use efficiency target level for each province and as the benchmark for the convergence target of each province.

### 3.2. Model Parameter Setting of the SSPs-HE Method

According to the results of the HE classification, this article divided the 31 provinces into four categories. In combination with the five socioeconomic pathways, the different convergence targets and convergence speed parameters were set for different types of HE in China. The qualitative and quantitative settings of the parameters are shown in the following tables (Table 2 and Table 3).

### 3.3. Verification of the Data Accuracy of the Total Water Use Forecast

**Figure 5:**
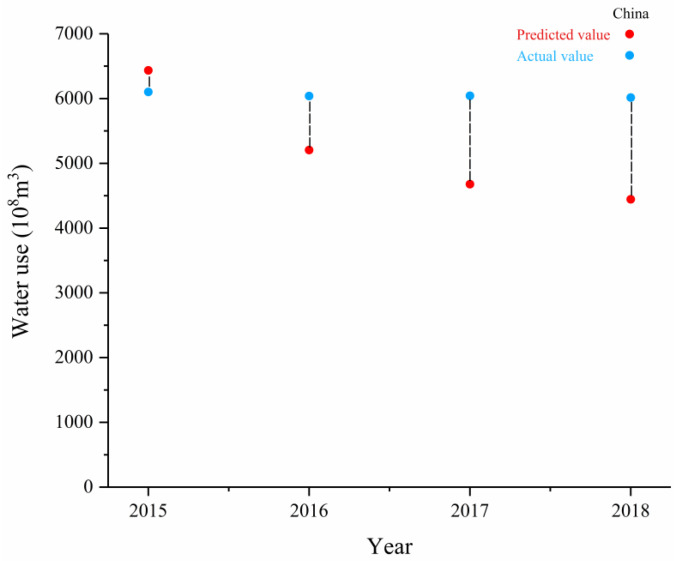
The predicted and actual values of China’s water use from 2015 to 2018.

A comparison of the prediction results and statistical data of water use in China and typical provinces from 2015 to 2018 was used to verify the simulation effectiveness of the conditional convergence model. To be consistent with the historical development pathway, the predicted values and actual values under the SSP2 scenario that would not significantly deviate from the social, economic, and technological trends obtained from the historical model were selected to verify each other. As shown in Figure 5, the actual values showed that the average water use in China from 2015 to 2018 was 6050.58 × 10^8^ m^3^, in 2015 it was 6103.2 × 10^8^ m^3^, and in 2018 it was 6015.5 × 10^8^ m^3^. During the same period, the average annual water use predicted by the model was 5190.94 × 10^8^ m^3^, in 2015 it was 6434.78 × 10^8^ m^3^, and in 2018 it was 4445.42 × 10^8^ m^3^. The average relative error of statistics and forecast data was 16.7%, the relative error range was 5–26%, and the relative error standard deviation was 12.2% (Figure 5).

**Figure 6:**
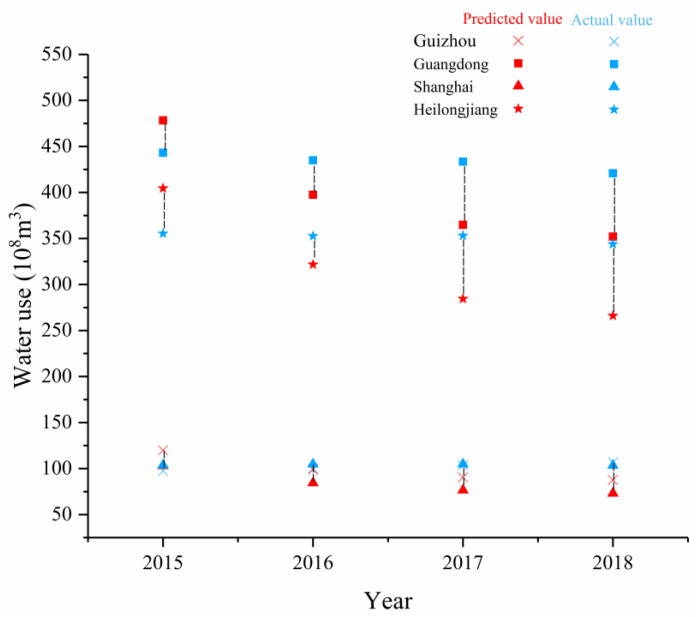
The predicted and actual values of water use in typical provinces from 2015 to 2018.

In addition, this study selected Guizhou, Guangdong, Shanghai, and Heilongjiang as representatives of the HE-1, HE-2, HE-3, and HE-4 regions to observe the degree of fit between the actual values and the predicted values. From 2015 to 2018, the actual values of the average annual water use of the four provinces of Guizhou, Guangdong, Shanghai, and Heilongjiang were 100.14 × 10^8^ m^3^, 427.44 × 10^8^ m^3^, 99.42 × 10^8^ m^3^, and 345.05 × 10^8^ m^3^ respectively. The average annual water use predicted by the model during the same period was 99.28 × 10^8^ m^3^, 398.18 × 10^8^ m^3^, 84.29 × 10^8^ m^3^, and 319.21 × 10^8^ m^3^, the average relative errors were 13.7%, 12.19%, 19.1%, and 16.18%, and the standard deviations of the relative errors were 15.74%, 9.82%, 11.2%, and 14.3%, respectively (Figure 6). The primary reason for the error was that the predicted values of GDP did not exactly match the actual values, and the error range was 7–8%. However, the water use efficiency only included water use for the agricultural, industrial, and service industries, and ignored artificial ecological environment water replenishment. The artificial ecological environment water replenishment in each province accounted for different proportions of the total water use, so the error range was relatively large.

### 3.4. Analysis of the Water Use Efficiency and Total Water Use Forecast Results

**Figure 7:**
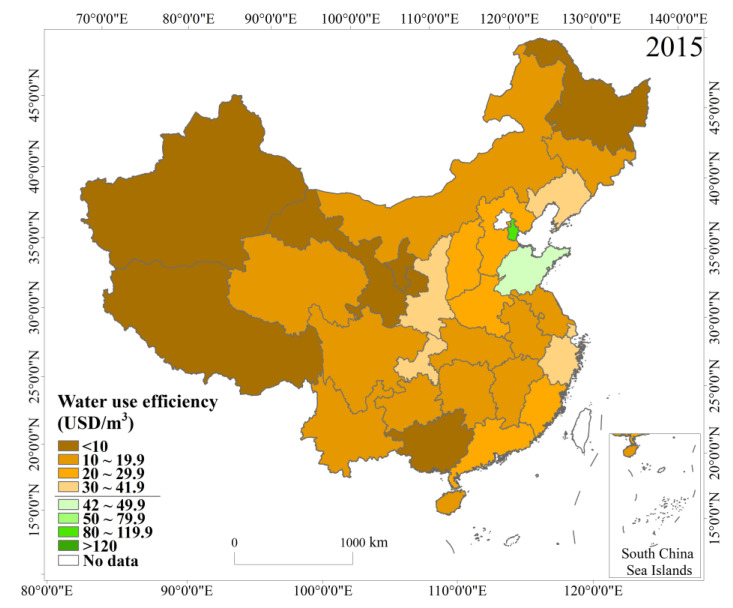
Spatial distribution of water use efficiency in each province in 2015.

As of the end of 2015, China’s water use efficiency was 18.74 USD/m^3^, and the water use was 6103.2 × 10^8^ m^3^. Among these figures, the service industry had the highest water use efficiency, reaching 69.04 USD/m^3^, the industrial water use efficiency was 38.59 USD/m^3^, and the irrigated agriculture water use efficiency was only 1.51 USD/m^3^. The water use efficiencies of northern China and eastern China were higher for the entire country, while the water use efficiencies of the northeast, northwest, and southwest were lower (Figure 7). China’s water use efficiency was higher than most developing countries, but it was far from the world’s advanced level in the same year. For example, the water use efficiency in South Korea was 46.50 USD/m^3^, Japan was 53.36 USD/m^3^, and the US was 40.67 USD/m^3^, which were much lower than the UK’s of 314.69 USD/m^3^ and Switzerland’s of 377.13 USD/m^3^.

**Figure 8:**
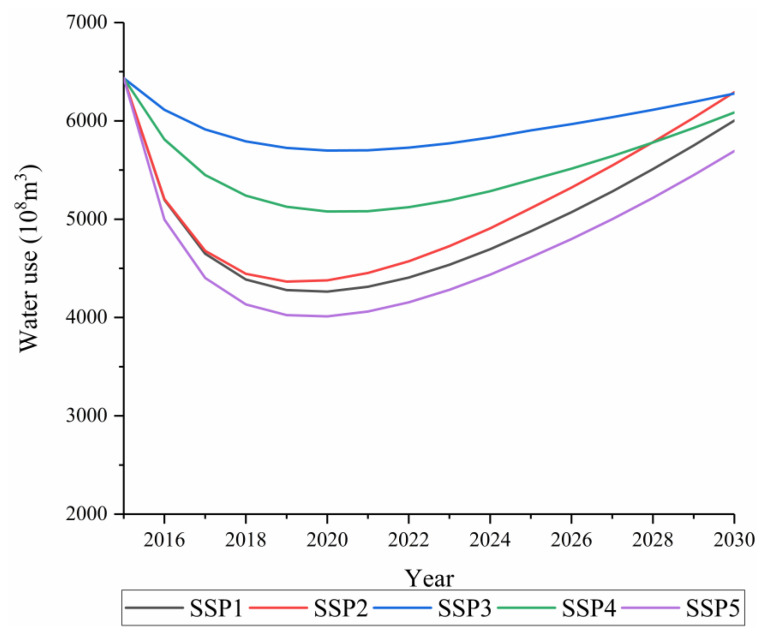
Trends of China’s total water use from 2015 to 2030 under the shared socioeconomic pathways (SSPs).

From the perspective of the evolution from 2016 to 2030 at the national level (Figure 8), there were significant differences in China’s total water use level between the different SSPs by 2030. The total water use level in China ranged from 5693.69 × 10^8^ m^3^ of SSP5 to 6292.11 × 10^8^ m^3^ of SSP2. In the publication “Views on the implementation of the strictest water resource management system”, the State Council set the goal of “establishing a red line for the development and utilization of water resources and controlling the total water use of the country within 7000 × 10^8^ m^3^ by 2030.” Therefore, water resource development and utilization control goals can be achieved under all the SSP pathways.

**Figure 9:**
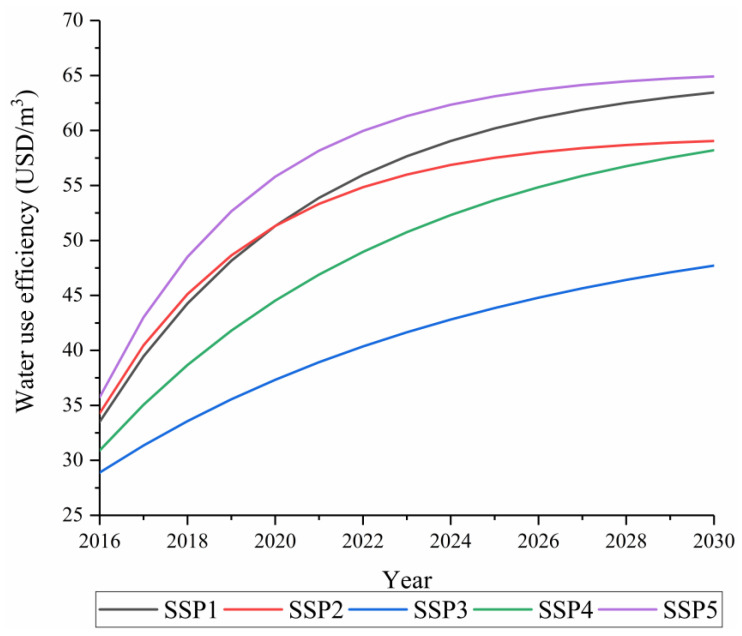
Trends in China’s water use efficiency from 2016 to 2030 under the shared socioeconomic pathways (SSPs).

By 2030, SSP5 will have the highest water use efficiency in the country, and water use will increase by 0.88 times compared with 2015, making it the most water-saving pathway among the five pathways. The SSP3 pathway showed the lowest water use efficiency and the slowest decline in water use, followed by SSP4. The water use efficiency of SSP1 and SSP2 displayed a medium growth rate, and the water use under SSP1 was the second lowest. In 2030, China’s total water use level will be 0.93 times that of 2015. The water use under SSP2 shows a rapid decline, but in the first few years close to 2030, the total water use under SSP2 displays a higher growth rate at the national level, primarily because it assumes a slower rate of convergence of the water use efficiency growth rate. In addition, SSP2 is more prosperous than SSP3, with fierce regional competition, and SSP4, with unequal development. Hence, it has the ability to develop water resources and provide water for more people. SSP1 was obviously better than SSP2 in controlling the growth of water use. From the perspective of the scale of total water use, the sustainable state was stronger (Figure 8 and Figure 9).

**Figure 10:**
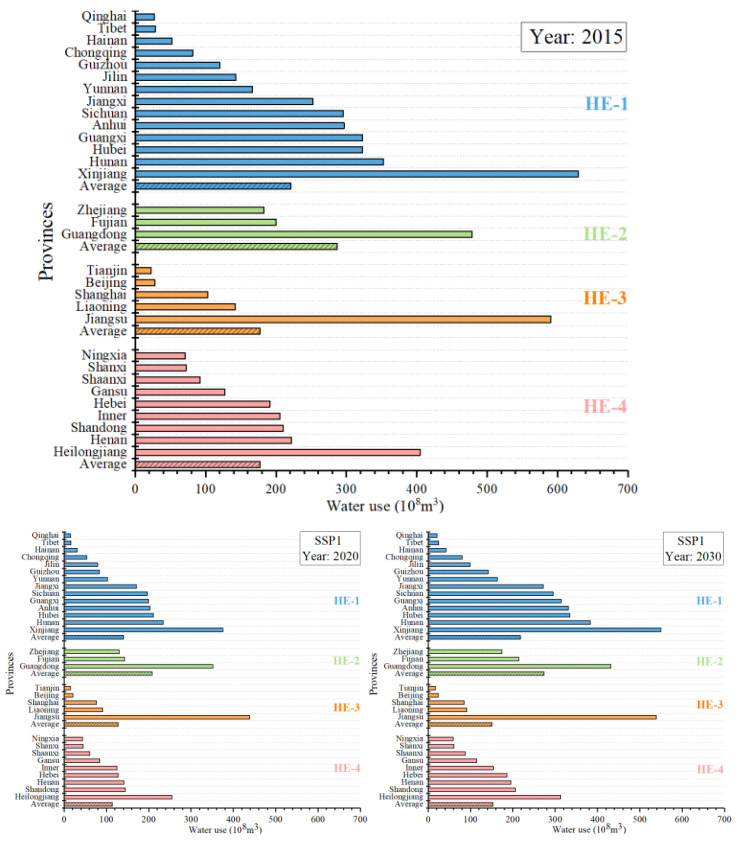

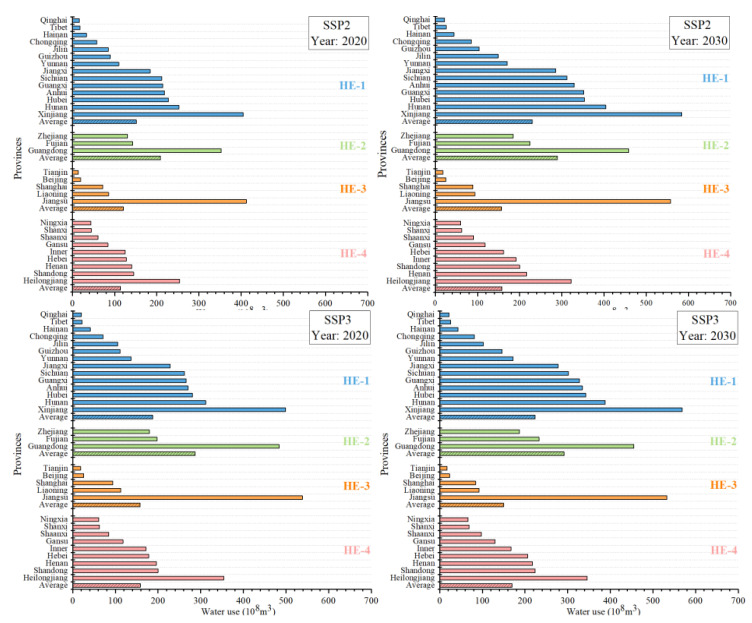

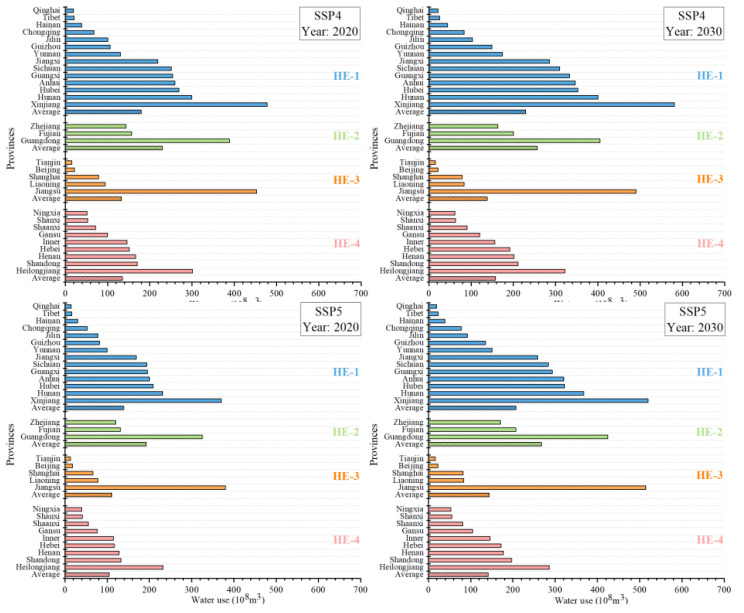
Water use in each province in 2015 and under the shared socioeconomic pathways (SSPs) in 2020 and 2030.

**Figure 11:**
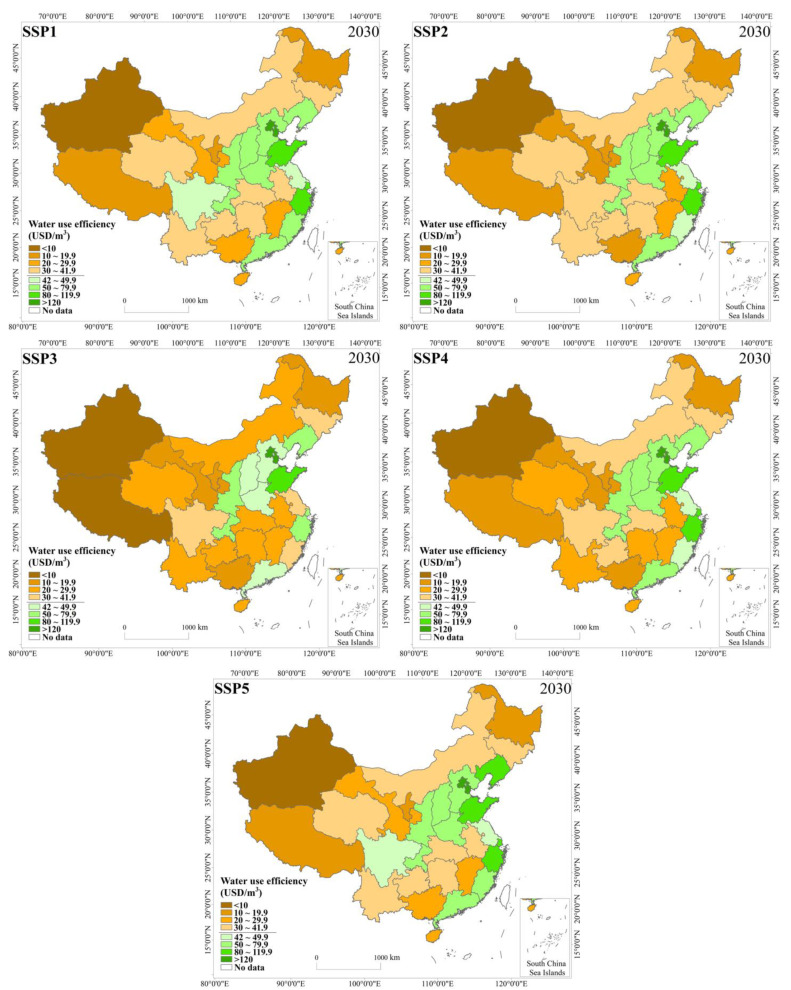
Spatial distribution of water use efficiency in each province under the shared socioeconomic pathways (SSPs) in 2030.

Figure 10 shows the changes in water use by provinces in the HE-1, HE-2, HE-3, and HE-4 areas under the different pathways in 2015, 2020, and 2030. Figure 11 shows the changes in water use efficiency by provinces in the HE-1, HE-2, HE-3, and HE-4 areas under the different pathways in 2030.

From the perspective of the evolution of the different types of provinces, the HE-3 areas with high water pressure but strong economic strength had high water use efficiency under the five pathways and could easily reach the national standard of 42 USD/m^3^ in 2030. Under SSP5, the water use efficiency was the highest. Under SSP1 and SSP4, there was still in a period of efficiency growth, and the stamina of the technological progress was relatively sufficient. Under SSP5, the improvement space for technological progress was relatively narrow compared with SSP1 and SSP4. Under SSP3, the water use efficiency converged slowly, and the water use efficiency was low overall. In addition, the technology lacked room for subsequent improvement. The area had insufficient water resources and good water-saving technologies. The average water use of HE-3 areas were the lowest among the four types of areas in 2030, and the SSP4 and SSP5 pathways had the lowest water use.

The HE-2 areas with low water pressure but strong economic strength generally had lower water use efficiency than the HE-3 areas. Since there was no high water pressure, the improvement in efficiency was limited. In SSP5, a development scenario based on fossil fuels, achieved the highest level of water use efficiency, followed by SSP1 and SSP4. However, SSP5 had the fastest improvement in the water use efficiency, and there was still much room for improvement in the future. Similar to SSP5, SSP1 and SSP4 reached China’s water use efficiency target value by 2030. SSP2 converged earliest, but the subsequent increase rate was low. The water use efficiency under SSP3 was very low and in a relatively stable state. Due to the low water pressure and strong economic strength, the HE-2 area had the largest average water use in 2030, and the SSP4 and SSP5 pathways had the lowest water use.

The HE-4 area ranked third in water use efficiency overall. Under SSP1 and SSP5, due to sufficient funds and open technologies, the water use efficiency was high, and there was a certain amount of room for improvement in the future, but the improvement was not large. Under SSP2, owing to the high water-saving pressure of the recent policies, the water use efficiency improved rapidly, but due to a lack of financial support and slow technological diffusion, the overall water use efficiency was not high. For the highly unbalanced SSP4 scenario, backward provinces, such as the HE-4 area, were in a disadvantaged position and lacked funds and talents. However, owing to the demonstration effect of the advanced provinces and the diffusion effect of the advanced technology to a certain extent, the water use efficiency slowly improved by partially catching up. The HE-4 area under SSP3 had basically stagnated in terms of water use efficiency. The HE-4 area had moderate average water use in 2030, with the lowest water use under the SSP1 and SSP5 pathways.

The overall water use efficiency was the lowest in the HE-1 area, and the water use was also low. Owing to the abundant water resources in the area, the motivation to improve the water use efficiency was limited. Poverty and no water pressure made nearly all provinces in the HE-1 area unable to meet the national water use efficiency targets under the various pathways in 2030. With the exception of the overall low water use efficiency, the other cases were similar to the HE-4 area.

### 3.5. Analysis of the Rebound Effect

According to the curves of water use efficiency and water use in China and each classified area (Figure 7, Figure 8, Figure 9, Figure 10 and Figure 11), this study found that under nearly all the pathways, the water use efficiency continued to increase. In addition, the water use curve first showed a decline and then a rising “U”-shaped posture. During the initial stage, the increase in the water use efficiency led to a decline in the total water use, which was related to the implementation of various water-saving measures, the development of irrigation technology, and the innovation of industrial water technologies [54]. However, with a continuous increase in the water use efficiency, the total amount of water use increased. This phenomenon shows that China’s future water use will have a rebound effect under the various shared socioeconomic pathways.

High water demand and water shortages are common problems faced by most countries. An important way for many countries to deal with the water crisis is to improve the irrigation efficiency (such as promoting new technologies to improve crop drip irrigation) and to allocate the water resources saved by agricultural water savings to industry, residents, and the ecological environment [13]. China is no exception. China is a large agricultural country. Since 1998, agricultural water has accounted for greater than 60% of the total water use, and irrigation water has accounted for 90% of the total agricultural water use. Irrigation water has always had problems, such as large water use, low water use efficiency, serious pollution, and obvious regional differences [55]. Currently, only 1.1% of rural residents in major irrigation areas have adopted water-saving technologies [56]. The improvements of water use efficiencies largely originate from improvements in the irrigation efficiency. However, the literature shows that increasing irrigation efficiency has a serious rebound effect [5,6,7,8]. For example, Song et al. [1] pointed out that due to technological progress in China, the increase in water use for agricultural production has offset the large amount of water resources saved by improving efficiency. Zhang et al. [57] conducted a quantitative study on the Tarim River Basin in northwest China and showed that the water resources saved by water-saving irrigation were not left in the river, but were reused to expand the irrigation of farmland, resulting in more water consumption. Liu et al. [58] used a structural decomposition analysis approach to explain the rebound effect of water-saving efforts in the Heihe River Basin in the arid area of northwest China, and they revealed that the virtual water export to a large extent offset the water saving efforts achieved by enhancing the water use efficiency in a river basin. Wang et al. [59] examined the largest water conservation irrigation area in the Tianshan region (northwest China), an arid area, to test the rebound effect on water conservation efforts in terms of its blue water footprint, which was also designated as irrigation water consumption. Currently, there are studies being conducted on largescale irrigation water use rebound issues and irrigation water consumption rebounds at the small scale [59]. Therefore, China’s future water use will experience a rebound effect, and the rebound effect of irrigation water use is inseparable. According to the simulation results of the SSPs, SSP3, which has backward technologies and an extremely low water use efficiency, had a greater impact on water resource utilization. Although the water use of each pathway reached the standard in 2030, the annual growth rate of water use was relatively high. This trend is not optimistic.

### 3.6. Defect Discussion

First, the Chinese government did not set a specific water use efficiency value in 2030 in the document the “Views on the implementation of the strictest water resource management system”. Therefore, the conditional convergence model lacks an accurate value for the target water use efficiency for 2030. To solve this problem, in this study, the target value was obtained using the method of trend extrapolation. Although this method is simple and clear, accuracy and reliability need to be considered. Second, this study used the method recommended by FAO to calculate the water use efficiency. In terms of composition, it lacks the consideration of artificial ecological environmental replenishment, which causes the error between the predicted value of the total water use and the statistical value of the total water use to be large.

## 4. Conclusions and Suggestions

Based on the SSPs framework combined with the water use efficiency accounting method recommended by the FAO and the HE classification method of the WFAS and according to the technology diffusion mechanism and conditional convergence model, this study predicted the water use efficiency and water use of 31 provinces in China from 2016 to 2030. The research conclusions are as follows:

(1) China’s total water use under the different SSPs has reached the strictest water resource management goal, but the development trajectory is quite different, and future water use will have a rebound effect. Under SSP1 and SSP5, the rate of increase in the water use efficiency in various places was found to be relatively rapid, and the total water use will reach a low state by 2030. The situation under SSP2 was different. The water use efficiency had the fastest convergence of all the pathways. However, when it converged to a certain level, it lacked the potential for subsequent development and remained stagnant. Therefore, with the growth of the GDP during the later period, the total water use will further increase. SSP3 was a bad situation. The water use efficiency has not been greatly improved for a long time, and the total water use remained high. SSP4 presented a highly unbalanced situation based on economic strength. The strong regions are getting stronger (such as the HE-2 regions and HE-3 regions), and the weak regions are getting weaker (such as the HE-1 regions and HE-3 regions). Hence, the water use efficiency is low, and the total water use is slowly increasing. In the future, China’s water use under the different SSPs will inevitably experience a rebound effect, which may be caused by the rebound effect of irrigation water use. The water use of SSP5 was the lowest, followed by SSP1. However, compared with SSP5 based on fossil fuel development, the SSP1 pathway of sustainable development can produce less greenhouse gas emissions and is more economical and environmentally friendly. Therefore, China should choose SSP1.

(2) Provinces with different HE categories displayed different overall water use conditions. Provinces belonging to HE-2 and HE-3 regions had higher water use efficiencies, while provinces in the HE-1 and HE-4 regions had lower efficiencies. It can be seen that the economic strength of the region, as compared with the endowment of water resources, had a greater impact on the water use efficiency. The HE-1 and HE-2 regions had higher water usages, while the HE-3 and HE-4 regions had lower water usages. This shows that the regional water resource endowment had a greater impact on water use than the economic strength. Provinces with different HE categories had the lowest water use efficiencies and water uses under the SSP5 pathway, but the SSP5 pathway posed too many environmental threats. It is necessary to consider the SSPs pathway selection in combination with the own economic strength and water resource endowments of the regions. Provinces under the HE-3 category had both the water-saving power and economic strength required for water-saving. The endogenous power of water-saving was relatively strong. Therefore, For HE-3 provinces, the SSP1 pathway was a better developmental pathway. The provinces under HE-4 face the dual pressure of fund shortages and a severe water use situation. They need to strive for the policy and fund support from the central government, and a special fund is essential to use money to save water. For these regions, the SSP2 pathway had a higher cost performance. Due to sufficient water resources but a lack of funds, the HE-1 areas can follow the development pathway of SSP1 and SSP5 and achieve water-saving objectives slowly over the long term. For HE-2 provinces, due to sufficient funds and safe water use, it should be committed to creating a harmonious relationship between maintaining human living standards and environmental water resources in the future development, which is suitable for the development pathway of SSP1.

Based on the above analysis, the following suggestions are proposed:

(1) Importance should be attached to technological progress. Although there is the presence of the rebound effect, it is undeniable that the level of water use efficiency still significantly reduces the initial total water use. Under the condition of ensuring GDP growth, although the total water use still increases, it is still under the total control target of the strictest water resource management system. More precisely, without an improvement in the technical efficiency, under the background of GDP growth, the total water use has already exceeded the total control target.

(2) The developmental concepts of coordination, openness, and sharing should be adhered to. China’s vast territory, complex national conditions, the degree of development varies from place to place, and inadequate and unbalanced development has become the primarily characteristics of the new era. Therefore, the implementation of water-saving technologies in all the regions should be adapted to the local conditions, according to their own development conditions, to achieve water-saving goals. At the same time, the principle of openness, inclusiveness, and interconnection among all regions should be adhered to so as to realize technological diffusion and benefit the backward areas.

(3) To alleviate the impact of the rebound effect on future water use, it is necessary to consider water-saving policies from the perspective of the rebound effect of irrigation water.

(4) At all scales, from farms to river basin scales, water resource accounts need to be established to conduct comprehensive water resource accounting and record and disclose changes in water resources at different scales. Additionally, the total amount of irrigated area needs to be controlled. Finally, value assessments need to be conducted to ensure that the public benefits generated by subsidizing efficiency improvements outweigh the costs.

## Figures and Tables

**Table 1:** Summary of the water use narrative scenarios under the shared socioeconomic pathways (SSPs) framework [21].

Pathway	Water Use Efficiency Level	Convergence Level	Convergence Speed	Scenario Description
SSP1	High	High	Low	All regions adhere to the development principles of openness, equality, mutual benefit, and technology spreads rapidly. Rapid urbanization in all regions in order to improve resource efficiency. The improvement of water resource utilization efficiency reflects that the water resource system is sustainable. The entire society has a good energy saving and emission reduction atmosphere.
SSP2	Medium	Medium	Very fast	The income growth of each region is moderate, and the process of urbanization is also moderate. Technological development is limited, and policies for environmental protection are limited. The improvement rate of the water use efficiency slowed down, barely reaching the planning target in 2030, and then technical progress was weak.
SSP3	Low	Low	Fast	Various regions are obviously divided and lack of coordination, leading to a closed and self-defense road. Technological development is in a conservative and stagnant state. High population growth, slow urbanization, and unscientific urban planning. The utilization rate of water resources is low and the water use is large
SSP4	Low (developing)/high (developed)	Medium	Medium	Due to the backward economic development and the limited investment in water-saving facilities, the water use efficiency is low in areas with low hydroclimatic complexity and low income. The areas with high incomes have higher water use efficiency under the support of strong economic strength. Under the dual pressure of backward economy and high complexity of hydroclimatic, water use efficiency in some areas is at a low level. The regional diffusion of technology in different levels of economic development.
SSP5	High	High	Very low	Rapid capital accumulation and large-scale greenhouse gas emissions. Construction of various water-saving projects to improve water use efficiency. Agro-ecosystems and water systems are highly managed and resource intensive.

**Table 2:** Qualitative description of the water use efficiency under the shared socioeconomic pathways (SSPs).

Pathway	Water Use Efficiency	HE-1	HE-2	HE-3	HE-4
SSP1	High	High	High-medium	High-low	High-medium
SSP2	Medium	Medium	Medium	Medium	Medium
SSP3	Low	Low-high	Low	Low-medium	low
SSP4	High (developed), low (developing)	Medium-low	Medium-high	Medium-high	Medium-low
SSP5	High	High	High	High	High

**Table 3:** Quantitative transformation of the convergence parameters for the simulation.

Pathway	HE-1		HE-2		HE-3		HE-4	
	Convergence Target (Multiple of Benchmark Target)	Convergence Time (Years)	Convergence Target (Multiple of Benchmark Target)	Convergence Time (Years)	Convergence Target (Multiple of Benchmark Target)	Convergence Time (Years)	Convergence Target (Multiple of Benchmark Target)	Convergence Time (Years)
SSP1	1.1	15	1.1	20	1.1	25	1.1	20
SSP2	1.0	15	1.0	15	1.0	15	1.0	15
SSP3	0.9	30	0.9	50	0.9	40	0.9	50
SSP4	1.0	30	1.1	30	1.1	30	1.0	30
SSP5	1.1	15	1.1	15	1.1	15	1.1	15

## Data Availability

The published statistical data comes from websites (http://www.fao.org/sustainable-development-goals/indicators/641/en/, http://www.stats.gov.cn/, and https://data.worldbank.org.cn/); for other data, please contact the corresponding author based on reasonable grounds.
